# *QuickStats*: Percentage* of Currently Employed^†^ Adults Aged ≥18 Years Who Received Influenza Vaccine in the Past 12 Months,^§^ by Employment Category^¶^ — National Health Interview Survey,** United States, 2012 and 2016

**DOI:** 10.15585/mmwr.mm6716a8

**Published:** 2018-04-27

**Figure:**
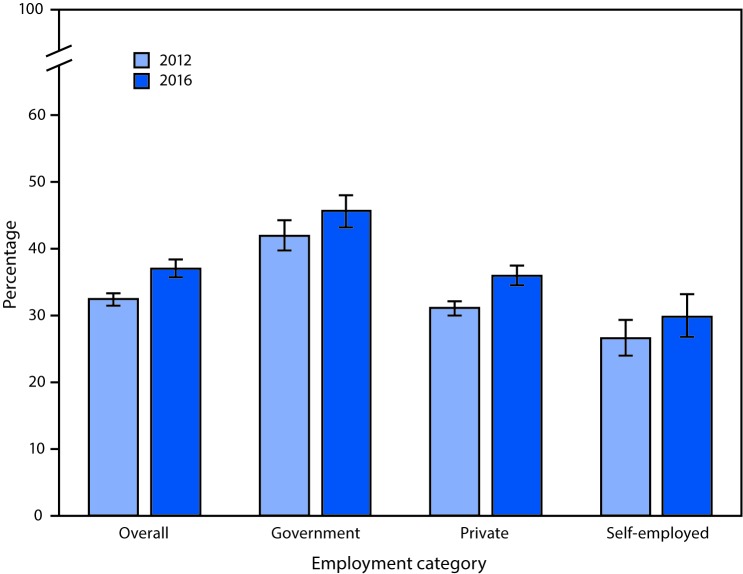
From 2012 to 2016, the percentage of employed adults who had received an influenza vaccine in the past 12 months increased overall (32.4% versus 37.0%), among government employees (42.0% versus 45.6%), and private-sector employees (31.1% versus 36.0%), but there was no significant increase among the self-employed (26.5% versus 29.8%). In both years, a higher percentage of government employees had received an influenza vaccine compared with private-sector employees, who had higher percentages than the self-employed.

**Source:** National Health Interview Survey, 2012 and 2016. https://www.cdc.gov/nchs/nhis.htm.

